# Localized spatial distributions of disease phases yield long-term persistence of infection

**DOI:** 10.1038/s41598-019-56616-3

**Published:** 2019-12-30

**Authors:** Promit Moitra, Sudeshna Sinha

**Affiliations:** Indian Institute of Science Education and Research Mohali, Knowledge City, SAS Nagar, Sector 81, Manauli, PO 140 306 Punjab India

**Keywords:** Biological physics, Statistical physics, thermodynamics and nonlinear dynamics

## Abstract

We explore the emergence of persistent infection in two patches where the phases of disease progression of the individuals is given by the well known SIRS cycle modelling non-fatal communicable diseases. We find that a population structured into two patches with significantly different initial states, yields persistent infection, though interestingly, the infection does not persist in a homogeneous population having the same average initial composition as the average of the initial states of the two patches. This holds true for inter-patch links ranging from a single connection to connections across the entire inter-patch boundary. So a population with spatially uniform distribution of disease phases leads to disease extinction, while a population spatially separated into distinct patches aids the long-term persistence of disease. After transience, even very dissimilar patches settle down to the same average infected sub-population size. However the patterns of disease spreading in the patches remain discernibly dissimilar, with the evolution of the total number of infecteds in the two patches displaying distinct periodic wave forms, having markedly different amplitudes, though identical frequencies. We quantify the persistent infection through the size of the asymptotic infected set. We find that the number of inter-patch links does not affect the persistence in any significant manner. The most important feature determining persistence of infection is the disparity in the initial states of the patches, and it is clearly evident that persistence increases with increasing difference in the constitution of the patches. So we conclude that populations with very non-uniform distributions, where the individuals in different phases of disease are strongly compartmentalized spatially, lead to sustained persistence of disease in the entire population.

## Introduction

Theoretical approaches, based on mathematical analyses and numerical simulations of models, have provided useful tools to gauge the influence of diseases in a population^1–3^. For instance, there have been interesting studies on disease control on networks through analysis of networks of sexually transmitted disease, analysis of transmission dynamics for Zika virus on networks and disease dynamics in multihost interconnected networks^4,5^.

Specifically, whether or not infection persists in the long-term in a population is an issue of much significance, and has potentially practical consequences for designing disease control policies. Now patterns of outbreaks such as spiral waves^6–8^ and self organized states characterized by scale-invariant behaviour emerging over a wide range of spatial and temporal scales^9^, as well as models focusing on the probability of infection transmission in networks^10–17^, have been extensively investigated. However the identification of features that determine the *long-term persistence* of infection, at time-scales that are large compared to a single disease cycle, is relatively unexplored. So the focus of our work here will be a particular issue that has immense bearing on the broad question of persistence of infection in a region: does the separation of a population into distinct sub-regions where individuals in different stages of the disease are spatially clustered, hinder or aid the long-term sustenance of disease? More generally, this line of enquiry has bearing on the broader question of how micro-structure of populations influences *asymptotic macrodynamics* of disease. That is, we focus on collective dynamical patterns that emerge in the long-term, from the interplay of local influences, such as continual self-sustained waves of infection in the population, along with complex temporal oscillations in the prevalence of infecteds. The results also have potential bearing on whether or not traditional analyses based on differential equations, typically built on the premise of well-mixed populations^18^, can capture all aspects of long-term persistence of infection. We will demonstrate in this work that our approach complements such traditional analyses, and provides additional insights determined crucially by the heterogeneity or “patchiness” of populations. This approach will shed light on important phenomena that are not captured by mean densities, and are very significantly affected by patchy distributions or spatial clustering of individuals in different stages of the disease.

Note that the focus of our work here is distinct from studies of the effect of network modularity on disease spreading^19^. Here the underlying connectivity is *not* modular. Rather the population lives on a two-dimensional plane, with regular nearest neighbours links. However, what we consider is a *structured distribution of the state variables*, i.e. a partitioned spatial distribution of the disease phases. So we consider long-term infection persistence in populations comprised of localized spatial patches with distinct distributions of the disease stages. Such spatial compartments can be considered as “communities”, characterized by different distributions of the disease stages among the individuals in each patch. The long-term impact of such initial non-uniformity in the spatial distribution of states is the key focus of our investigation.

## Model

The SIRS cycle is one of the most successful models that have captured the essence of the epidemiology of infectious diseases in mathematical terms^20,21^. It is particularly relevant for modelling the progression of communicable diseases that are non-fatal or where the mortality rates are not significant enough to be included in the model, and where the immunity wanes over time so that individuals can get reinfected. This class of diseases involve a stage of temporary immunity, and include diseases such as tuberculosis, influenza, typhoid, tetanus, cholera and small pox^22,23^. For instance, North American influenza dynamics, in particular the H5N1 and H1N1 strains of the influenza virus, have been well described by the SIRS model^24^.

The SIRS cycle involves three distinct disease stages. To begin with, an individual is *susceptible* to infection. This stage denoted by the symbol S. When the susceptible individual comes into physical contact with another infected individual in its neighborhood, the susceptible person becomes infected. This *infectious* stage is denoted by the symbol I. So a susceptible individual will progress from S to I, after such a contact. Note that the contact with an infected individual is a stochastic process occurring with a probability determined by the composition of the population. After the infectious stage is a *refractory* stage, which represents the temporary immunity from the disease. It is denoted by the symbol R. In this stage, people are immune to further infection, and also do not have the capacity to infect other individuals in its neighbourhood. Since this immunity is only temporary, an individual in the refractory stage becomes susceptible again after a while. So the refractory period is followed by the susceptible stage again. Hence the disease progression is cyclic.

In this work the SIRS cycle will be described by a cellular automaton (CA), as proposed in ref. ^25^. The discrete time steps in the cellular automaton model will be denoted by an integer *t*. The population lives on a 2-dimensional plane, and the location of each individual is given by indices (*i*, *j*) on a 2-dimensional square lattice. The *phase* in the disease cycle of each individual at location (*i*, *j*) is given by an integer counter *τ*_*i*,*j*_(*t*), which takes values from 0, 1, …, *τ*_0_, with *τ*_0_ = *τ*_*I*_ + *τ*_*R*_, where *τ*_*I*_ is the length of the infectious period, *τ*_*R*_ is the length of the refractory period, and *τ*_0_ + 1 is the total disease cycle length^25–28^.

The identification of the stage of disease (S, I or R) by the variable *τ* is given as follows:

(i) When *τ*_*i*,*j*_(*t*) = 0, the individual at site (*i*, *j*) is susceptible (S);
(ii) When 1 ≤ *τ*_*i*,*j*_(*t*) ≤ *τ*_*I*_, the individual at site (*i*, *j*) is infected (I);
(iii) When *τ*_*i*,*j*_(*t*) > *τ*_*I*_, the individual at site (*i*, *j*) is in the refractory period (R).

The dynamics of the disease phases are as follows: When phase *τ*_*i*,*j*_(*t*) ≠ 0, i.e. the individual is either infected or refractory (I or R), the counter updates by 1 every time step. This goes on till the refractory period ends, after which the individual becomes susceptible again. This mathematically implies that when *τ*_*i*,*j*_(*t*) = *τ*_0_, at the next time step it becomes zero, i.e. *τ*_*i*,*j*_(*t* + 1) = 0. This signifies that after completion of the R stage the individual goes back to stage S. So the cellular automaton model of the cycle *S* → *I* → *R* → *S* is given concisely by the following update rules^25,27^:

1 $$\begin{array}{llll}{\tau }_{i,j}(t+\mathrm{1)} & = & {\tau }_{i,j}(t)+1 & {\rm{if}}\,1\le {\tau }_{i,j}(t) < {\tau }_{0}\\  & = & 0 & \,{\rm{if}}\,{\tau }_{i,j}(t)={\tau }_{0}\end{array}$$

We consider the refractory stage to be longer than the infective period, i.e. *τ*_*R*_ > *τ*_*I*_, which is a typical situation in many classes of disease. Specifically in this work, without significant loss of generality, we take ${\tau }_{I}=4$, ${\tau }_{R}=9$ and *τ*_0_ $={\tau }_{I}+{\tau }_{R}$ = 13.

### Infection spreading

We consider that a susceptible individual (i.e. with *τ* = 0) will become infected *if one or more individuals in its nearest neighbourhood is in the infected stage*. So the condition for infection spreading is as follows: if *τ*_*i*,*j*_(*t*) = 0 and even one neighbouring site is such that 1 ≤ *τ*_*x*,*y*_(*t*) ≤ *τ*_*I*_, then *τ*_*i*,*j*_(*t* + 1) = 1. Here {*x*, *y*} are the site indices of the four nearest neighbours of site (*i*, *j*) on the 2-dimensional lattice: (*i* − 1, *j*), (*i* + 1, *j*), (*i*, *j* − 1) and (*i*, *j* + 1). The initial fraction of infecteds, susceptibles and refractory individuals in a patch is denoted by *I*_0_, *S*_0_ and *R*_0_ respectively. In this work the full range of *I*_0_ is explored, with *S*_0_ = *R*_0_.

A significant feature of the disease dynamics here is the interplay of stochastic and deterministic processes. The transition from susceptible to infected, is governed by the disease stages of the individals in the neighbourhood, and so it depends crucially on the specific random initial configuration of the different disease phases amongst the individuals. Since the pattern of disease progression is determined by the random initial state, different probability distributions of disease phases in the population may yield significantly different long-term dynamics. However the disease cycle, once initiated, is completely deterministic, going over the infective and refractory periods in *τ*_0_ time steps and returning back to the susceptible stage. Since the SIRS cellular automata has a finite state space, the full state of a population under disease progression is necessarily recurrent. When all individuals are susceptible, the infection dies out, i.e. all susceptibles is an absorbing state of the system^29^. Lastly note a significant difference from certain earlier models here. In our system, no set of agents are kept artificially in the infected state as in refs. ^25,26^, and so there is the possibility of complete extinction of disease.

We now investigate the spread of infection in a group of spatially distributed individuals, where at the individual level the disease progresses in accordance with the SIRS cycle given above. In this work we focus on an unexplored aspect of such systems, namely we attempt to ascertain the *dependence of the persistence of infection on the structure of the population*. So the specific question of relevance here will be the correlation between sustained long-time persistence of disease in the two patches and the difference in the initial states of the two patches. That is, we will investigate the dependence of infection persistence (if any) on the initial differences between the patches constituting the population.

In particular, we consider a population of individuals in two distinct patches. Each patch is a 2-dimensional *N* × *N* lattice where every node, representing an individual, has 4 nearest neighbors. Within each patch, the phases of the disease cycle are randomly distributed among individuals such that the distributions of infecteds, susceptibles and refractory individuals are spatially uniform over the lattice constituting each spatial patch. The boundaries of the patches are fixed, with no interactions outside the patch. So each population patch mimics a closed region, such as an island or an isolated habitat.

We first recall the principal results for a single patch where the disease phases have a uniform random distribution^27^. Here persistent infection emerges only when the initial population consists of an admixture of susceptible and refractory individuals, and there is atleast one infectious seed. There is a window of persistence in *I*_0_, with *I*_0_ → 0 at the lower end of this window, i.e. when the initial population has a uniform distribution of susceptible and refractory individuals, a single contagious agent can give rise to sustained infection. On the other hand there is a critical fraction of initial infecteds *I*_0_ ($\sim 0.2\mbox{--}0.3$) beyond which the system cannot sustain infection in the long-term. So counter-intuitively too many initial infecteds (approximately more than a third of the population) also leads to extinction of the infection.

Now we consider two such patches, with each patch characterized by distributions of infected, susceptible and refractory individuals, which can be very different. Such a separation in terms of the initial composition provides insight for situations where the spatial distribution of the disease phases have become highly non-uniform, and this could occur for instance in cases of attempted containment or movement restrictions. The patches are connected through a small number of links. The connections between the two patches may be spatially adjacent or randomly located. The fraction of inter-patch links along the adjoining edges of the patches is denoted by *f*_*ic*_. This quantity is analogous to a connection density between the patches, and reflects the probability that individuals from a patch can interact, through contact, migration or transport across the boundary, with a set of individuals in the other patch. In this work we will consider a wide range of connection densities, from one or two connections, to links along the entire edge of the boundary between the patches. The central results of our work here, based on order parameters obtained by averaging over space and time, do not depend on the location of the links.

## Spatiotemporal Patterns of Disease Spreading

We first study the spatiotemporal patterns of the spread of infection in the two patches connected via a few links along an adjoining edge. With no loss of generality, we display results for two patches of size 100 × 100.

In order to explore the effect of the non-uniformity of the patches on persistence of disease, which is the principal focus of our investigation, we consider the patches to have varying initial fractions of susceptibles ${S}_{0}^{(i)}$, infecteds ${I}_{0}^{(i)}$ and refractory individuals ${R}_{0}^{(i)}$, where *i* = 1,2 is the index of the community. That is, the patches are comprised of random admixtures of infected, susceptible and refractory individuals, which may differ on an average, in varying degrees. So the patches are distinct in terms of the disease stages of the individuals present in the patch, and the non-uniformity of the patches is in the distribution of the state variables in the patch.

**Figure 1:**
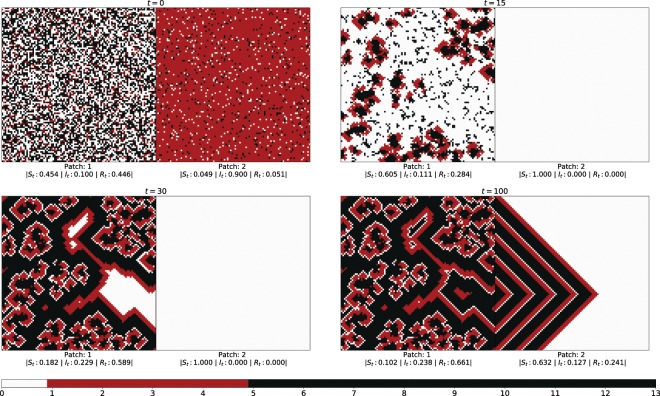
Infection spreading patterns for two patches, where the initial fraction of infecteds is ${I}_{0}^{(1)}=0.1$ in patch 1 and ${I}_{0}^{(2)}=0.9$ in patch 2, with the initial fraction of susceptible and refractory individuals being equal in both patches (i.e. ${S}_{0}^{(i)}={R}_{0}^{(i)}$ for *i* = 1, 2). Here ${\tau }_{I}=4$ and ${\tau }_{R}=9$, and the fraction of boundary sites that have inter-patch connections is *f*_*ic*_ = 0.1, i.e. 10 sites on an average interact with the other patch.

**Figure 2:**
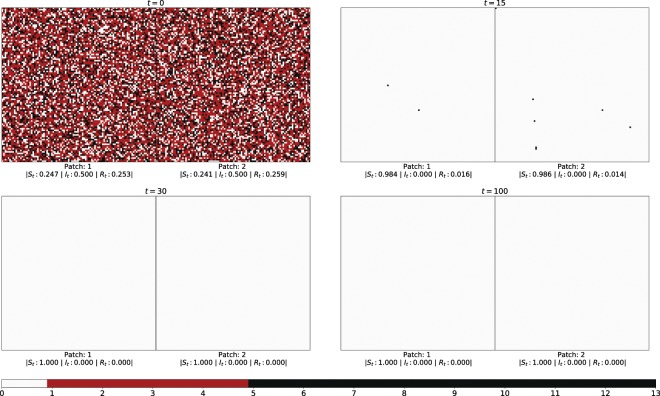
Infection spreading patterns for two patches, where the initial fraction of infecteds is ${I}_{0}^{\mathrm{(1)}}={I}_{0}^{\mathrm{(2)}}=0.5$ in both patches, and ${S}_{0}^{\mathrm{(1)}}={S}_{0}^{\mathrm{(2)}}={R}_{0}^{\mathrm{(1)}}={R}_{0}^{\mathrm{(2)}}$. Here ${\tau }_{I}=4$ and ${\tau }_{R}=9$, and all sites along adjoining edge of the two patches have inter-patch connections (i.e. *f*_*ic*_ = 1.0).

Specifically we explore the number of infecteds in the emergent state of the entire population, under varying differences in the initial states of the patches. That is, we investigate the long-term presence of infection in both patches under progressively increasing difference in the initial states of the patches. Representative results are displayed in Figs. 1 and 2. Figure 1 shows the infection spreading patterns for two patches, where the initial fraction of infecteds is ${I}_{0}^{(1)}=0.1$ in one patch and ${I}_{0}^{(2)}=0.9$ in the other. As a reference Fig. 2 shows the infection spreading patterns for two patches, where the initial composition is identical. Note that the *average* initial fractions of infected, susceptibles and refractory individuals is the same in both systems, i.e ${I}_{0}^{(1)}=0.1$ in Fig. 2 is equal to $\frac{({I}_{0}^{\mathrm{(1)}}+{I}_{0}^{\mathrm{(2)}})}{2}$ in Fig. 1. So the populations in the two figures do not differ on an average. However, the populations are very different in terms of the “patchiness” of the spatial distribution of the infecteds. In Fig. 1 the patches are significantly different in initial composition and so the system as a whole is strongly non-uniform, while in Fig. 2 the disease phases are uniformly distributed across the population and there is no difference in the state distributions of the two spatial patches. It is clearly evident then from Fig. 1 vis-a-vis Fig. 2, that *populations partitioned into distinct patches yield long-term persistence of disease, while a population with uniformly distributed individuals in different stages of disease, results in the extinction of the infection from the entire population*.

Specifically then, we reiterate, persistent infection refers to the situation where infection is always present somewhere in the spatial patch, at all points in time, for time-scales that are several orders of magnitude larger than a disease cycle. So when infection is persistent, it never dies out, in the very long times over which the system is monitored, and some level of infection is always maintained in the system. Further the complicated patterns of disease-spreading repeat over time, resulting in complex periodic oscillations in the population of infected individuals, which never goes to zero. In contrast, when the infection does not persist, the number of infecteds in the entire system quickly goes to zero, and remains zero for all time henceforth, in the absence of external perturbations. So the pattern of disease progression is determined by the random initial state, and different spatial distributions of disease phases in the initial population yield different long-term dynamics. However, if persistently infected, each individual has a cyclic disease progression pattern, since the disease cycle is deterministic with time period *τ*_0_. So in the case of persistent infection the entire population exhibits complex patterns comprised of cyclic patterns of the individuals whose phases are determined by the initial random spatial configuration. If one views the entire population as a very high-dimensional dynamical system, persistent infection arises when the initial state of the system is attracted to complex oscillations. On the other hand, if the initial state is attracted to an uniform all-susceptible state (where *τ* = 0 for all individuals) the infection dies out.

**Figure 3:**
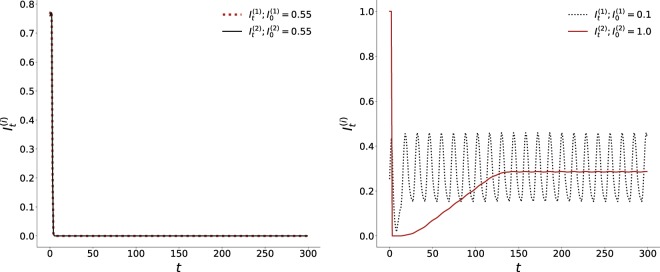
Time evolution of the fraction of infected individuals *I*_*t*_^(*i*)^ in two patches, of size *N* = 100 × 100, where the initial fraction of infecteds is (left) ${I}_{0}^{\mathrm{(1)}}={I}_{0}^{\mathrm{(2)}}=0.55$ and (right) ${I}_{0}^{\mathrm{(1)}}=0.1$ and ${I}_{0}^{\mathrm{(2)}}\mathrm{=1.0}$ (with ${S}_{0}^{(i)}={R}_{0}^{(i)}$ for *i* = 1,2). On an average two boundary sites have inter-patch connections (i.e. *f*_*ic*_ = 0.02). Note that in the left panel we have identically distributed patches, with the same average number of infecteds as the average of the two patches in the right panel, i.e. $\frac{({I}_{0}^{\mathrm{(1)}}+{I}_{0}^{\mathrm{(2)}})}{2}=0.55$ in both panels. Clearly, the infected fraction rapidly goes to zero when the phases of disease are uniformly distributed among the patches, while persistent infection emerges, with average infected fraction around 0.3, when the patches have very different local compositions, even though the average composition of the entire population is identical for both cases. Note that we have followed the system up to 10^4^ time steps, and these temporal trends hold even when the system is tracked to time-scales an order to magnitude larger than the ones presented here.

**Figure 4:**
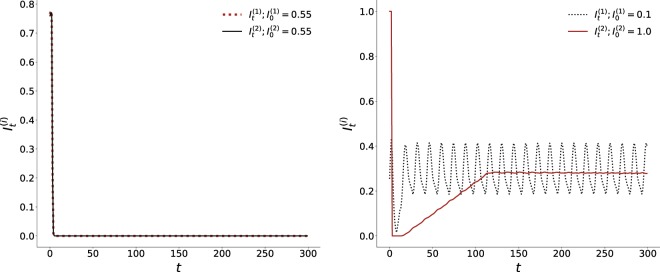
Time evolution of the fraction of infected individuals ${I}_{t}^{(i)}$ in two patches, of size *N* = 100 × 100, where the initial fraction of infecteds is (left) ${I}_{0}^{\mathrm{(1)}}={I}_{0}^{\mathrm{(2)}}=0.55$ and (right) ${I}_{0}^{\mathrm{(1)}}=0.1$ and ${I}_{0}^{\mathrm{(2)}}=1.0$ (with ${S}_{0}^{(i)}={R}_{0}^{(i)}$ for *i* = 1,2). Note that in the left panel we have identically distributed patches, with the same average number of infecteds as the average of the two patches in the right panel, i.e. $\frac{({I}_{0}^{\mathrm{(1)}}+{I}_{0}^{\mathrm{(2)}})}{2}=0.55$ in both panels. Here all sites along the adjoining edge of the patches have connections across the boundary in the other patch (i.e *f*_*ic*_ = 1), with the inter-patch links being spatially random. Clearly again, the infected fraction rapidly goes to zero when the phases of disease are uniformly distributed among the patches, while persistent infection emerges, with average infected fraction around 0.3, when the patches have very different local compositions, even though the average composition of the entire population is identical for both cases.

Figures 3 and 4 shows the time evolution of the fraction of infected individuals in the two patches for the case of patches with identical average initial states, and for patches with very different initial states. It is clear from the time series that when the initial state of the patches are close, the wave forms are similar in both amplitude and frequency. However, when the initial states of the patches are markedly different, the time evolution of the infected sub-set has *very different amplitude*, though same frequency. Interestingly, the *average fraction of infecteds is the same in both patches, though the pattern of evolution is significantly different*. The patch which had a much higher initial fraction of infecteds evolves to an oscillatory pattern with very low amplitude around the average value of close to 1/3, while the fraction of infecteds in the patch with low initial fraction of infecteds oscillates with large amplitude around the same mean value. So after transience, two patches with very different initial average composition settle down to identical average behaviour. Nevertheless, the local evolution of infection bears a discernible mark of the distinct initial states. So while the average number of infecteds of the two distinct patches evolve to the same mean value, the amplitudes of the oscillations about the mean are significantly different.

So if a patch has an initial composition such that in isolation infection persists in it, on coupling with any other patch it exhibits the same behaviour, i.e. infection will be sustained in the patch irrespective of the composition of the coupled patch and the density of inter-patch links. However, if a patch does not sustain infection in the long-term, then on coupling with another patch two scenarios emerge. First, if the other patch too cannot yield persistent infection in isolation, then on coupling the infection dies out in both patches. But remarkably, if the second patch has sustained infection when isolated, it will drive the infection to persist in the long-term in *both* patches. This is interesting, as the same would not occur if one had two patches with the same distribution, having the same average composition as the case of the two distinct patches. In case of uniform distribution of disease phases in the patches, and consequently homogeneous distribution in the full population, the infection would die out in both patches. Only when the patches are *sufficiently different* does the entire system sustain infection in the long run. This suggests the first important result: *well-mixed populations, though commonly used as models, cannot capture the long-term persistence of infection in a region*.

## Dependence of the Persistence Order Parameter on Heterogeneity

In order to quantify the long-term persistence of disease we use a persistence order parameter 〈〈*I*_*t*_〉〉 defined in refs. ^27,30^. This quantity is the fraction of infected individuals in the entire population, averaged over time of the order of several disease cycles (after long transients), and further averaged over a large sample of random initial conditions. This quantity indicates the absence of infection in the long-term when it is equal to zero, and indicates sustained presence of infection when non-zero. So it can serve as a good indicator of long-term persistence of infection in a population, and can help in quantitatively identifying transitions to persistent infection.

We first consider the initial infected fraction ${I}_{0}^{\mathrm{(1)}}$ of one patch to be ${I}_{0}^{\ast }$ = 0.1. This implies that initially, 10% of the population in the patch is infected. The fractions *S*_0_ = *R*_0_ for both patches, unless otherwise specified. Now, if this patch was considered independent of the second patch, this fraction of initial infecteds ${I}_{0}^{\ast }$ would yield persistent infection^27^. The initial infected fraction of the second patch ${I}_{0}^{\mathrm{(2)}}$^)^ is varied as ${I}_{0}^{\ast }+\Delta$. So Δ serves as an useful parameter reflecting the difference between the initial fractions of infected individuals present in the patches, and is indicative of the localized structure of the spatial distribution of disease stages in the population. In particular, in our representative examples, Δ ∈ [0.0, 0.9]. This provides us a parameter reflecting the spatial compartmentalization, or non-uniformity, of the disease phases within the system. Larger Δ indicates a more non-uniform spatial distribution, with the constituent patches being more diverse.

**Figure 5:**
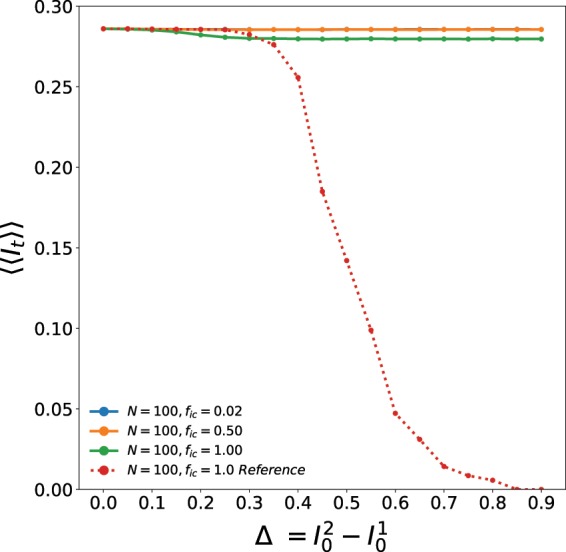
Dependence of the persistence order parameter 〈〈*I*_*t*_〉〉 of patch 2 (i.e. average asymptotic fraction of infected individuals in the second patch) on parameter Δ (which quantifies the difference in the initial composition of the patches). Here the initial infecteds ${I}_{0}^{\mathrm{(1)}}={I}_{0}^{\ast }$ and ${I}_{0}^{\mathrm{(2)}}={I}_{0}^{\ast }+\Delta$, patch size is 100 × 100 and the fraction of boundary sites with inter-patch connections is: *f*_*ic*_ = 0.02 (blue), 0.5 (yellow) and 1.0 (green). The red dotted curve shows the variation of persistence order parameter 〈〈*I*_*t*_〉〉 for the reference case where the two patches were not segmented, and behave as a single uniform patch, with the infected individuals initially distributed uniformly, with ${I}_{0}^{\mathrm{(1)}}={I}_{0}^{\mathrm{(2)}}=\langle {I}_{0}\rangle ={I}_{0}^{\ast }+\frac{\Delta }{2}$.

We now estimate the time and ensemble averaged persistence order parameter 〈〈*I*_*t*_〉〉, i.e. the asymptotic fraction of persistent infected individuals in the entire population comprised of the two patches. As mentioned earlier, this quantity signals the absence of infection in the long-term when it is equal to zero, and indicates sustained presence of infection when non-zero. Figure 5 shows the dependence of 〈〈*I*_*t*_〉〉 on increasing differences between the initial states of the two patches Δ. We scan the full range of Δ between 0 to 0.9. When Δ = 0, both patches have equal initial fractions of infected individuals $({I}_{0}^{\mathrm{(1)}}={I}_{0}^{\mathrm{(2)}}={I}_{0}^{\ast }=\mathrm{0.1)}$, and so the spatial distribution of disease phases of the entire population is uniform, and the system is effectively a single patch. When Δ is very large, for instance equal to 0.8, ${I}_{0}^{\mathrm{(2)}}={I}_{0}^{\ast }+\Delta \mathrm{=0.1}+\mathrm{0.8=0.9}$. This implies that the second patch has a much higher density of infected individuals than the first one, thus yielding an exceedingly non-uniform spatial distribution of disease phases in the system. The question we focus on here is the correlation between this spatial heterogeneity in disease stages and the long-term behaviour of the coupled patches, specifically in terms of the sustained presence of disease and the patterns of infection spreading.

Also note that as Δ is varied, the average fraction of initially infected individuals in the full system, comprised of both the patches, also changes. In particular, as we vary Δ from 0.0 to 0.9, with ${I}_{0}^{\mathrm{(1)}}=0.1$, ${I}_{0}^{\mathrm{(2)}}$ varies from 0.1 to 1.0. This implies that the collective average infected fraction of the two patches $\langle {I}_{0}\rangle =\frac{{I}_{0}^{\mathrm{(1)}}+{I}_{0}^{\mathrm{(2)}}}{2}=\frac{{I}_{0}^{\ast }+{I}_{0}^{\ast }+\Delta }{2}={I}_{0}^{\ast }+\frac{\Delta }{2}$, varies from (0.1 + 0.1)/2 = 0.1 to (0.1 + 1.0)/2 = 0.55. In order to establish that the emergent patterns of infection in the two coupled patches, depend on the initial spatial non-uniformity of the individual disease phases, and not on the collective average infection initially present in the patches, it is illustrative to compare our observations in two contrasting conditions. As a reference for comparison (as we had done earlier in Figs. 1 and 2), we first find the asymptotic fraction of infecteds in the entire population, 〈〈*I*_*t*_〉〉, for a population comprised of two patches whose average initial state is identical, i.e. there is spatial uniformity (on an average) in the entire population. Further we consider the initial infected fraction for the reference case to be the same as the average value of the infecteds in the non-uniform case, i.e. ${I}_{0}^{(1)}={I}_{0}^{(2)}={I}_{0}^{\ast }+\Delta /2$ for the reference case. Since this case considers uniform distribution over both patches, with the same average value of infecteds as the non-uniform cases, it provides a good baseline to identify features arising solely from the non-uniformity of spatial distributions, and not average properties.

So by comparing the results arising in the case of two distinct patches, with the reference curve for the uniform case, we can establish the marked impact of spatial non-uniformity on the long-term persistence of infection (cf. Fig. 5). It is clearly evident from the figure that when the initial spatial distribution of infected individuals in the two patches is significantly different, the infection is persistently present in both the patches, for all initial conditions. The contrast with the case of one uniform patch is evident through the reference curve, where the *persistence of infection drops drastically* as the fraction of initial infecteds ${I}_{0}^{\ast }+\frac{\Delta }{2}$ increases. However, when the two patches have distinct densities of initial infection as in Fig. 1, with the sum total of infecteds in the two patches being the same as in the reference system on average, *the infection sustains itself in both the patches*. Thus we can conclude that the presence of infection in the long-run originates due to the non-uniform spatial distribution of the disease phases in the two patches, and not due to the variation in the overall average initial infection present in the system.

**Figure 6:**
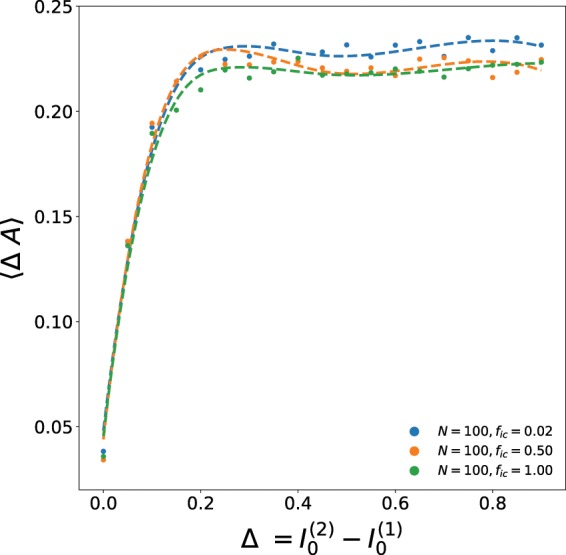
Dependence of the average difference in amplitudes of the emergent oscillations in the infected sub-population of the two patches 〈Δ*A*〉, on Δ (which quantifies the difference in the initial composition of the patches). Here the initial infecteds ${I}_{0}^{\mathrm{(0)}}={I}_{0}^{\ast }$ and ${I}_{0}^{\mathrm{(2)}}={I}_{0}^{\ast }+\Delta$, patch size is 100 × 100, and the fraction of boundary sites with inter-patch connections *f*_*ic*_ is 0.02, 0.5 and 1.0.

Figure 6 shows the dependence of the average difference in amplitudes of the emergent oscillations in the size of the infected sub-population in the two patches 〈Δ*A*〉, on the difference in the initial composition of the patches, quantified by parameter Δ. As in Fig. 5, here the initial infecteds ${I}_{0}^{\mathrm{(0)}}={I}_{0}^{\ast }$ and ${I}_{0}^{\mathrm{(2)}}={I}_{0}^{\ast }+\Delta$. Different fractions *f*_*ic*_ of boundary sites with inter-patch connections are investigated. It is clear that there is a sharp transition to a large amplitude difference in the oscillatory patterns of the two patches as Δ → 0. This suggests that the smallest non-uniformity in the constituent patches yields *distinct temporal patterns*, even though the *average* quantities (such as the average number of infecteds, susceptibles and refractory individuals) evolve to *same* values in the two patches after short transience. So, as evident through the spatiotemporal spreading patterns in Fig. 1, the *initial differences in the constitution of the patches is clearly discernible even at long-times, in spite of the homogenization of the average composition of the communities*.

**Figure 7:**
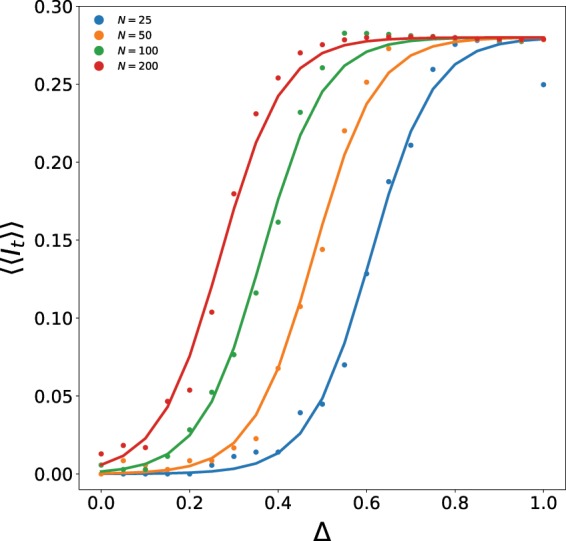
Dependence of the persistence order parameter 〈〈*I*_*t*_〉〉 of patch 2 on parameter Δ, which determines the initial infecteds of the two patches as follows: ${I}_{0}^{\mathrm{(1)}}={I}_{0}^{\ast }-\frac{\Delta }{2}$ and ${I}_{0}^{\mathrm{(2)}}={I}_{0}^{\ast }+\frac{\Delta }{2}$, with ${I}_{0}^{\ast }$ = 0.5. So Δ is a parameter that quantifies the difference in the initial composition of the patches, while maintaining the same average fractions of disease phases in the entire population, i.e. *I*_0_^(2)^ − *I*_0_^(1)^ = Δ, with $\frac{({I}_{0}^{\mathrm{(1)}}+{I}_{0}^{\mathrm{(2)}})}{2}\mathrm{=0.5}$ across all Δ. Here patch size *N* varies from 25 to 200, and *f*_*ic*_ = 1. The data is fitted to the sigmoidal logistic function $\frac{{I}_{max}}{1+{e}^{-(\Delta -{\Delta }_{mid})/b}}$, with the following best fit values: *I*_*max*_ = 0.28, *b* = 0.07, and Δ_*mid*_ = 0.61 for *N* = 25, Δ_*mid*_ = 0.48 for *N* = 50, Δ_*mid*_ = 0.36 for *N* = 100, Δ_*mid*_ = 0.27 for *N* = 200.

Figure 7 shows the dependence of the persistence order parameter 〈〈*I*_*t*_〉〉 of patch 2 (i.e. average asymptotic fraction of infected individuals in the second patch) on parameter Δ, where the initial infecteds of the two patches is given in terms of Δ as follows: ${I}_{0}^{\mathrm{(1)}}={I}_{0}^{\ast }-\frac{\Delta }{2}$ and ${I}_{0}^{\mathrm{(2)}}={I}_{0}^{\ast }+\frac{\Delta }{2}$, with ${I}_{0}^{\ast }$ = 0.5. So Δ is a parameter that quantifies the difference in the initial composition of the patches, while maintaining the same average fractions of disease phases in the entire population, i.e. ${I}_{0}^{\mathrm{(2)}}-{I}_{0}^{\mathrm{(1)}}=\Delta$, with $\frac{({I}_{0}^{\mathrm{(1)}}+{I}_{0}^{\mathrm{(2)}})}{2}=0.5$ across all Δ. Results from different patch sizes are shown, ranging from patches of size 25 × 25 to 200 × 200. Since the persistence of infection does not depend in any significant way on the densities of inter-patch links, and the transition curve is almost invariant under variation of *f*_*ic*_, only the illustrative value of *f*_*ic*_ = 1 is displayed in the figure. The first striking feature is that there is a *sharp transition to persistent infection when the difference between the patches is sufficiently large*. The data from different patch sizes and inter-patch connectivities all fit a sigmoidal form well, i.e. the dependence of the persistence order parameter on Δ goes as

$$\langle \langle {I}_{t}\rangle \rangle =\frac{{I}_{max}}{1+{e}^{-(\Delta -{\Delta }_{mid})/b}}$$

Here *I*_*max*_ gives the value of the persistence order parameter 〈〈*I*_*t*_〉〉 where the curve saturates after the transition, Δ_*mid*_ indicates the value of Δ where the transition curve crosses the midpoint value of 〈〈*I*_*t*_〉〉, and *b* reflects how steeply the transition curve rises, with small *b* indicating a very sharp rise. Our best fits to this form yield *b* = 0.07 clearly indicating a sharp transition. *I*_*max*_ = 0.28 for all system sizes and inter-patch connection densities, and indicates that in the state with persistent infection, the fraction of infecteds is close to 1/3. The value of Δ_*mid*_ reflects the minimum degree of non-uniformity of the patches necessary to yield a transition to the persistent state. We observe that the value Δ_*mid*_ goes to zero as a power law, as system size *N* increases: ${\Delta }_{mid} \sim {N}^{-0.4}$. This observation implies that larger patches yield persistent infection at smaller Δ, and suggests that one obtains persistent infection for very small differences in the constituent patches when the patches are very large.

## Conclusion

In summary,we have explored the long-term persistence of infection qualitatively and quantitatively in two patches, where the disease progression of the individuals was given by the SIRS model and an individual became infected on contact with another infected individual. Such weakly connected islands or patches of habitats can provide a test-bed to study the sustenance of disease in adjacent regions^31^.

Our central result is the following: if a population is structured into distinct patches, the infection will persist. This is in contrast to the situation where there is a uniform admixture of infected, refractory and susceptible individuals in a region, in which case there will be rapid transient waves of infection that will quickly die out. While there is not enough controlled data currently to apply the model directly to real-life, we anticipate that such numerical simulations will provide incentive to study the persistence of infection in controlled spatial patches.

Importantly, from the perspective of theoretical modelling, a consequence of our results is the following: we have established that the long-term persistence of infection is crucially dependent on the spatial structure and variability of the initial distribution of disease phases, and is not merely determined by the average properties. This implies that descriptions of disease spreading that are relevant to well-mixed populations, such as the widely employed differential equation based models, does not adequately capture infection persistence in a region, thus providing impetus to go beyond spatially averaged models.
